# A Systematic Review on the Occurrence of Antimicrobial-Resistant *Escherichia coli* in Poultry and Poultry Environments in Bangladesh between 2010 and 2021

**DOI:** 10.1155/2023/2425564

**Published:** 2023-02-01

**Authors:** Md. Saiful Islam, Md. Jannat Hossain, Md. Abdus Sobur, Sadia Afrin Punom, A. M. M. Taufiquer Rahman, Md. Tanvir Rahman

**Affiliations:** ^1^Department of Microbiology and Hygiene, Faculty of Veterinary Science, Bangladesh Agricultural University, Mymensingh 2202, Bangladesh; ^2^Department of Microbiology and Public Health, Faculty of Veterinary, Animal and Biomedical Sciences, Khulna Agricultural University, Khulna 9100, Bangladesh; ^3^Naogaon District Hospital, Naogaon 6500, Bangladesh

## Abstract

Antimicrobial resistance (AMR) is a significant public health issue in Bangladesh like many other developing countries where data on resistance trends are scarce. Moreover, the existence of multidrug-resistant (MDR) *Escherichia coli* exerts an ominous effect on the poultry sector. Therefore, the current systematic review, following the Preferred Reporting Items for Systematic Reviews and Meta-Analyses (PRISMA) guidelines, was conducted to find out the AMR scenarios in *E. coli* isolates sourced from poultry and poultry environments in Bangladesh between 2010 and 2021. Following the PRISMA guidelines, a total of 17 published scientific articles were selected for this systematic review. This review revealed that 18 out of 64 districts in Bangladesh reported *E. coli* in poultry, having a higher prevalence (combined prevalence: 69.3%, 95% confidence interval, CI: 67.3-71%). Moreover, the prevalence ranged from 24.3% to 100%. This review found that *E. coli* isolates showed resistance to 14 antimicrobial classes and 45 different antimicrobial agents, including the last-line (reserve group) antibiotics and banned antimicrobial categories for the treatment of infections in agricultural animals. Phenotypic resistance of *E. coli* against penicillins and beta-lactamase inhibitors (20.2%-100%), cephalosporins (1.9%-100%), fluoroquinolones (5.98%-100%), aminoglycosides (6%-100%), tetracyclines (17.7%-100%), carbapenems (13.6%-72.7%), macrolides (11.8%-100%), polymyxins (7.9%-100%), phenicols (20%-97.2%), sulfa drugs (44.7%-100%), cephamycins (21.4%-48.8%), nitrofurans (21.4%-63.2%), monobactams (1.2%), and glycylcyclines (2.3%) was recorded in the last decades in Bangladesh. Also, 14 articles reported MDR *E. coli* in poultry, including a 100% MDR in nine articles and a 92.7% (95% CI: 91.2-94%) combined percentage of MDR *E. coli* isolates. Twenty-four different AMR genes encoding resistance to beta-lactams (*bla*_TEM_, *bla*_CTX-M-1_, *bla*_CTX-M-2_, *bla*_CTX-M-9_, *bla*_OXA-1_, *bla*_OXA-47_, *bla*_SHV_, and *CITM*), colistin (*mcr1* and *mcr3*), fluoroquinolones (*qnrB* and *qnrS*), tetracyclines (*tetA*, *tetB*, and *tetC*), sulfonamides (*sulI* and *sulII*), trimethoprim (*dfrA1*), aminoglycosides (*rmtB*), streptomycin (*aadA1*), gentamicin (*aac-3-IV*), erythromycin (*ereA*), and chloramphenicol (*catA1* and *cmlA*) were detected in *E. coli* isolates. The presence of MDR *E. coli* and their corresponding resistance genes in poultry and poultry environments is an alarming issue for all health communities in Bangladesh. We suggest a regular antimicrobial surveillance program with a strong One Health approach to lessen the hazardous effects of AMR *E. coli* in poultry industries in Bangladesh.

## 1. Introduction

Bangladesh has a population of more than 165 million people, making it one of the most densely populated nations in the world [1]. The poultry industry in Bangladesh has grown into a prosperous agricultural industry [2]. Poultry farming in rural and urban regions is rapidly expanding to provide regular protein needs and financial support to a large population [3]. According to reports, this industry provides 22%-27% of the country's total meat supply [4, 5]. Bangladesh has more than 3.7 million poultry population, which contributes to the country's gross domestic product (GDP) [6]. Poultry farming also acts as a self-income facility for the younger generation rather than searching for jobs.

Different types of poultry species are being reared in Bangladesh, such as broilers, layers, turkeys, guinea fowl, quails, ducks, geese, and pigeons. Most poultry farmers lack knowledge of proper farming systems and antibiotic usage. As a result, there is expanding concern with respect to the potential for the emergence and transmission of antimicrobial resistance (AMR) to humans through the food supply chain and environmental components [7].

*Escherichia coli* is a Gram-negative, facultative anaerobic, rod-shaped bacterium in the family of *Enterobacteriaceae*. It is generally found in the gastrointestinal tracts of poultry, humans, and other animals [8]. Most of the strains of *E. coli* are nonpathogenic; however, there is concern that some strains of *E. coli* could spread from animals to humans and even cause illness in commercially farmed chickens [9]. Strains that lead to food poisoning include enteropathogenic *E. coli* (EPEC), enterotoxigenic *E. coli* (ETEC), enteroinvasive (EIEC), and enterohemorrhagic *E. coli* (EHEC) [10]. Pathogenic *E. coli* has an adverse effect on hosts with a compromised immune system [11]. Moreover, various strains of *E. coli*, including enterohemorrhagic *E. coli* O157:H7, Shiga toxin-producing *E. coli*, and others, show devastating impacts on humans by causing different zoonotic diseases, such as enteritis, meningitis, endocarditis, urinary tract infections, septicemia, hemolytic-uremic syndrome, and epidemic diarrhea, in humans [12]. In the case of poultry, it causes yolk sac infection, omphalitis, cellulitis, swollen head syndrome, coligranuloma, and colibacillosis [13].

The spread of AMR is the most divisive issue in the health of humans, animals, and ecosystems in the twenty-first century [14, 15]. The spread of AMR has also emerged as a significant barrier to economic development [16]. The overuse and misuse of antibiotics play a significant role in the emergence and spread of antibiotic-resistant *E. coli*, which can be transmitted to humans through food or direct contact with sick animals [17]. Antibiotics are widely used in poultry rearing as growth stimulants or to treat infectious diseases [18, 19]. A development in AMR is inevitable because of the widespread use of antibiotics in both clinical and nonclinical settings in developing countries like Bangladesh [20]. Bacteria including *E. coli* have developed multidrug resistance (MDR) due to the haphazard way in which antibiotics are used [21]. Increased morbidity, death, and healthcare expenditures may result from the emergence of MDR strains to antimicrobial therapies [22]. In the last 20 years, the emergence of MDR strains has increased dramatically. Food-producing animals and their products have been identified as a source of resistance genes [23]. The resistance genes in *E. coli* are acquired through selective pressure, induction, or mutation [24]. Bacterial AMR genes can be spread horizontally and vertically to other bacteria, and they can also infiltrate the human food chain [25].

MDR *E. coli* in poultry is a serious public health concern. Many of the MDR *E. coli* also have zoonotic potentiality. They have the potential to spread to humans and other One Health components. Many of these AMR determinants can also be transmitted to other human bacterial pathogens via transferable genetic materials. In this systematic review, we have focused on the occurrence, distribution, and patterns of AMR in *E. coli* in poultry, their environment, and their reservoirs and modes of transmission, as well as the transmission of *E. coli* in poultry, and the prevention and control strategies of *E. coli* infections in poultry to update our knowledge for adopting effective intervention strategies and better management systems to reduce AMR-related hazards linked to poultry in Bangladesh.

## 2. Materials and Methods

### 2.1. Review Strategies

The review was conducted in accordance with the standard methods for systematic reviews, which were outlined in the Preferred Reporting Items for Systematic Reviews and Meta-Analyses (PRISMA) [26]. According to PRISMA guidelines, the following steps were taken during the review: (1) possibly pertinent articles were categorized using database search, (2) evaluation of how applicable the articles are to the review, (3) evaluation of the quality of relevant articles, and (4) data extraction, screening, and analysis.

### 2.2. Source of Information and Search Strategies

We utilized a methodical approach for the purpose of identifying published articles that reported the occurrence of anti-microbial-resistant *E. coli* in poultry in Bangladesh. We performed a written survey using PubMed, Web of Science, ScienceDirect, Google Scholar, ResearchGate, and Crossref databases to find out published articles (between 2010 and 2021) on the antimicrobial-resistant *E. coli* situation in Bangladesh's poultry health division. During the survey, we downloaded the relevant articles using the Bangladesh Agricultural University Library Network (http://catalog.bau.edu.bd). The study's goals informed the creation of a set of Boolean keywords such as “AND” (for words of a category) and “OR” (for words within a category). We have broken down the search phrases into four distinct groups: outcomes, populations, descriptors, and regions. While deciding on the final list of articles, the following criteria were undertaken: (1) identical articles were checked and eliminated from consideration, (2) those articles that did not fulfill the inclusion criteria that had been set were eliminated, (3) articles that did not fit our aim and scope were not considered, and (4) articles published before 2010 were eliminated. Words used to search databases were “Antimicrobial resistance”, “Antibiotic-resistant”, “AMR”, “Multidrug-resistant”, “MDR”, “*Escherichia coli*”, “*E. coli*”, “Poultry”, “Broilers/Layers”, “Turkey”, “Ducks”, “Poultry farms”, “Poultry farm environments”, “Bangladesh”, and “*E. coli* in poultry in Bangladesh”. Moreover, we imposed no limitations on either the language used or the kinds of articles published. We looked at every title and abstract that was at our disposal.

### 2.3. Data Screening

We screened all of the retrieved published articles to include them in our review. In order to be considered for review, studies had to satisfy the following criteria:

- Only those peer-reviewed articles were included in which *E. coli* was detected by polymerase chain reaction (PCR) assay
- All peer-reviewed studies describing the topic of antimicrobial-resistant *E. coli* in poultry in Bangladesh which were studied and published between January 2010 and December 2021
- All articles describing the prevalence or occurrence or characterization of antimicrobial-resistant *E. coli* in Bangladesh
- The studies were included if they examined the prevalence of antimicrobial-resistant *E. coli* and used a selective screening method to identify them (culture or molecular approach) as a first diagnostic step (i.e., directly from the samples)

### 2.4. Outcomes

The occurrence of antimicrobial-resistant *E. coli* in the specified samples was the major or primary result. The secondary outcomes include the classes of antimicrobials, antimicrobial-resistant genotypes, and the occurrence of multidrug-resistant *E. coli*.

### 2.5. Data Extraction

Using both primary and secondary findings as criteria, two authors (M.S.I. and M.J.H.) simultaneously determined which papers were included in the review. Each author used their own copy of a standard form to extract the data. The extracted data were then double-checked, and any discrepancies were ironed out through a round of author-wide discussion. After extraction, all the data were recorded according to different categories such as study areas, study period, types of poultry, sample types and size, number of samples, presence or absence of *E. coli*, percentages (also numbers) of sensitivity and resistance to various antibiotics, detection methods for *E. coli* and antibiotic susceptibility, citation, name of the first author, and publication year.

### 2.6. Data Analysis

For the statistical analysis, all the extracted data were initially incorporated and sorted in Excel-365 (Microsoft/Office 365, Redmond, Washington, USA). Subsequently, sorted data were then exported to GraphPad Prism (Prism 8.4.2, San Diego, California, USA) to get prevalence. Moreover, in order to calculate a prevalence estimate, we used GraphPad Prism to generate a binomial 95% confidence interval (CI) using the Wilson/Brown Hybrid technique [27]. In order to determine the total number of articles, descriptive data were compiled. Moreover, ArcMap software version 10.7 (ArcGIS Enterprise, ESRI, Redlands, CA, USA) was used to plot a map showing the study areas and the spatial distribution of antimicrobial-resistant *E. coli* in poultry in Bangladesh. The forest plot maps of the prevalence of *E. coli* with their MDR profiles extracted from selected published articles were prepared using Excel-365 after calculating the prevalence and 95% CI by GraphPad Prism software.

## 3. Results

### 3.1. Overall Data Acquisition

**Figure 1:**
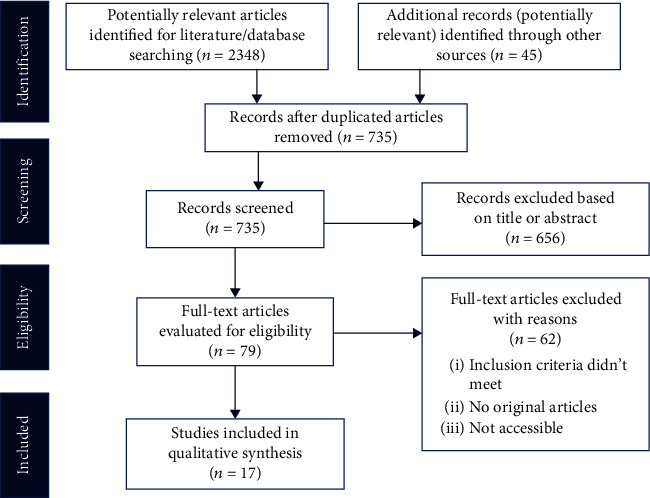
Flow diagram of PRISMA guidelines showing the search and selection process of published articles between 2010 and 2021.

A total of 2348 articles were identified during the initial screening. During the database search, 45 additional articles were retrieved. After eliminating duplicates, 735 distinct articles resulted. When determining whether or not a report was duplicated, we looked at whether or not it contained the same information in the fields for authors, published year, title, volume, issue, and page number of the articles. Following the removal of the articles that did not satisfy the eligibility requirements, the remaining 17 articles were finally selected for extracting and analyzing data (Figure 1 and Table 1).

These articles were published between 2015 and 2021, with the study year spanning from 2012 to 2020 (Table 1). In these 17 articles, sample categories include broilers, layers, and turkeys; sample types include cloacal swabs, feces, poultry dressing, egg surface swabs, chicken meat, internal organs (liver, lung, intestine, cecum, trachea, egg yolks, etc.), and poultry environmental samples (poultry pen, liter, soil, air, feed, water, farm sewage, etc.) (Table 1).

**Figure 2:**
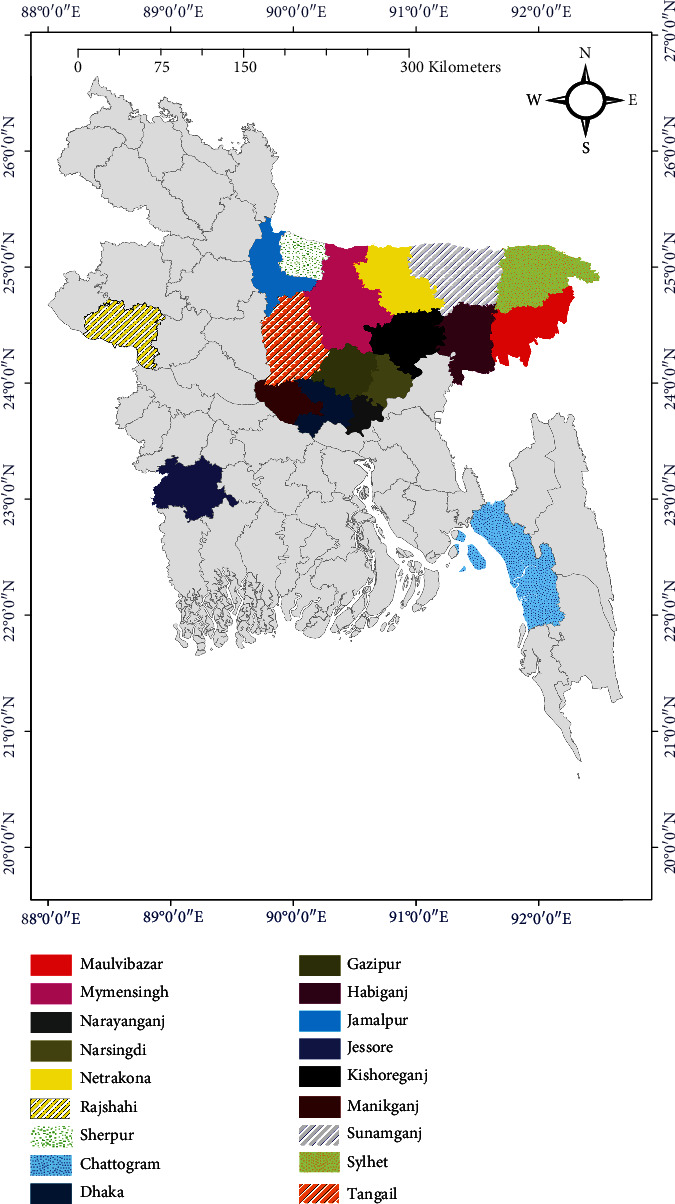
Map showing the study areas and the spatial distribution of antimicrobial-resistant *E. coli* in poultry in Bangladesh (based on published articles between 2010 and 2021). The area map was prepared using ArcMap 10.7.

Among the 64 districts of Bangladesh, research studies on antimicrobial-resistant *E. coli* were focused in 18 districts, of which most of the studies were conducted in Mymensingh, Dhaka, Sylhet, and Chattogram districts because of the easy access to samples and research laboratories in those districts (Figure 2). These studies were conducted either in an individual district or a combination of two or more districts of Bangladesh (Table 1). However, the study district of one study [7] was unknown.

### 3.2. Overall Prevalence of *E. coli* in Poultry

**Figure 3:**
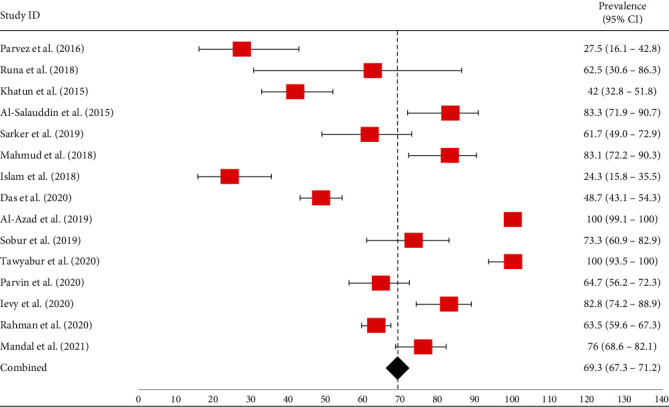
Forest plot of *E. coli* prevalence isolated from different poultry samples. The red-colored square dot represents point estimates of *E. coli* with their 95% confidence intervals, whereas the black-colored diamond-shaped point represents the combined *E. coli* prevalence acquired from selected published articles between 2010 and 2021 in Bangladesh. The forest plot was created with Excel-365 after calculating prevalence and 95% confidence interval using GraphPad Prism software.

Using analysis, overall, the combined prevalence of *E. coli* isolates sourced from poultry and poultry environments was 69.3% (95% CI: 67.3-71.2%) (Figure 3). The prevalence of *E. coli* in broilers ranged from 24.3% (95% CI: 15.8-35.5%) to 100% (99.1-100%) (Table 1 and Figure 3). In layers, the lowest prevalence was 61.3% (184/300, 95% CI: 55.7-66.7%), and the highest was 82.8% (95% CI: 74.2-88.9%) (Table 1 and Figure 3). Moreover, two articles [7, 36] did not mention any prevalence or occurrence rate of *E. coli* isolates in the case of layer samples; they only researched on 104 and 392 *E. coli* isolates, respectively (Table 1). The prevalence of *E. coli* in turkey was 100% (95% CI: 93.5-100%) (Figure 3).

### 3.3. Antimicrobial Resistance Profiles of *E. coli* in Poultry

According to 17 selected articles, *E. coli* sourced from poultry and poultry environments were found to be phenotypically resistant to 14 different classes of antibiotics (including 45 types of antibiotics), such as penicillins, cephalosporins, fluoroquinolones, cephamycins, carbapenems, polymyxins, monobactams, aminoglycosides, tetracyclines, sulfa drugs (folate pathways inhibitors), phenicols, macrolides, glycylcyclines, and nitrofurans (Table 2). All the articles recorded resistance of *E. coli* isolates to two or more antibiotics.

#### 3.3.1. Resistance to Penicillins and Beta-Lactamase Inhibitors

In the case of penicillins, the antibiotic ampicillin showed high resistance in *E. coli* isolates, with a prevalence ranging from 73.7% to 100% (95% CI ranged from 56.6% to 100%), 100% (95% CI ranged from 56.6% to 100%) to penicillin, 83.3%-100% (95% CI ranged between 71.9% and 100%) to amoxicillin, 20.2%-41.9% (95% CI ranged between 13.9% and 52.4%) to the combination of amoxicillin and clavulanate acid, and 28.6%-70.9% (the range of 95% CI was 1.7%-79.4%) to the combination of piperacillin and tazobactam (Table 2).

#### 3.3.2. Resistance to Cephalosporins and Carbapenems

*Escherichia coli* isolates showed resistance to various cephalosporin antibiotics, including 2.3%-100% (95% CI ranged from 0.4% to 100%) to ceftriaxone, 53.5%-100% (95% CI ranged from 43% to 100%) to cefotaxime, 1.8%-57.1% (95% CI ranged between 0.3% and 78.6%) to ceftazidime, 46.5%-100% (95% CI ranged between 36.4% and 100%) to cephalexin, and 72.1%-85.7% (the range of 95% CI was 60.1-97.5%) to cefepime. *E. coli* isolates also exhibited resistance to cefixime, cephradine, cefuroxime, and cefaclor (Table 2). Moreover, *E. coli* isolates showed lower to higher resistance to carbapenems (imipenem, prevalence: 13.6%-65.8%, 95% CI: 6.4%-73.9%; meropenem, prevalence: 41.9%-72.7%, 95% CI: 29.7%-82.7%) classes of antibiotics (Table 2).

#### 3.3.3. Resistance to Fluoroquinolones

*Escherichia coli* isolates from poultry and poultry environments in Bangladesh showed resistance to fluoroquinolones, such as ciprofloxacin (prevalence: 6% to 100%, 95% CI: 1.6%-100%), levofloxacin (prevalence: 22.2%-83%, 95% CI: 9%-90.3%), nalidixic acid (prevalence: 61.6%-100%, 95% CI: 51.1%-100%), norfloxacin (prevalence: 5.98%-50%, 95% CI: 0.3%-65.5%), gatifloxacin (prevalence: 38.9%-50%, 95% CI: 20.3%-61.4%), pefloxacin (prevalence: 61.1%-88.4%, 95% CI: 38.6%-93.6%), and ofloxacin (prevalence: 55.6%-56.9%, 95% CI: 33.7%-75.4%) (Table 2).

#### 3.3.4. Resistance to Aminoglycosides

*Escherichia coli* isolates exhibited resistance to aminoglycosides class of antibiotics, e.g., 6% to 100% (95% CI ranged from 1.6% to 100%) to gentamicin, 16.4%-100% (95% CI: 6.2%-100%) to streptomycin, and 27.9%-100% (95% CI: 19.5%-100%) to neomycin (Table 2).

#### 3.3.5. Resistance to Tetracyclines, Macrolides, and Polymyxins

*Escherichia coli* sourced from poultry showed a multifarious degree of resistance to tetracyclines (tetracycline, prevalence: 17.7%-100%, 95% CI: 12.8%-100%; oxytetracycline, prevalence: 93%-100%, 95% CI: 85.6%-100%; doxycycline, prevalence: 68.6%-78.9%, 95% CI: 58.2%-85.4%); macrolides (erythromycin, prevalence: 16.2%-100%, 95% CI: 7.7%-100%; and azithromycin, prevalence: 11.8%-34.9%, 95% CI: 2.1%-45.4%) and polymyxins (colistin, prevalence: 10.5%-100%, 95% CI: 4.4%-100%, and polymyxin B, prevalence: 7.9%-8.1%, 95% CI: 3.9%-15.9%) (Table 2).

#### 3.3.6. Resistance to Other Classes of Antibiotics

*Escherichia coli* sourced from poultry and poultry environments exhibited resistance to other classes of antibiotics, such as phenicols (chloramphenicol, prevalence: 20%-97.2%, 95% CI: 11.6%-99.9%), sulfa drugs/folate pathway inhibitors (sulfamethoxazole-trimethoprim, prevalence: 44.7%-100%, 95% CI: 35.9%-100%, and sulfonamides, prevalence: 44.7%, 95% CI: 35.9-53.9%), cephamycins (cefoxitin, prevalence: 21.4%-48.8%, 95% CI: 7.6%-59.2%), nitrofurans (nitrofurantoin, prevalence: 21.4%-63.2%, 95% CI: 7.6%-71.5%), monobactams (aztreonam, prevalence: 1.2%, 95% CI: 0.1-6.3%), and glycylcyclines (tigecycline, prevalence: 2.3%, 95% CI: 0.4-8.1%) (Table 2).

### 3.4. Multidrug Resistance Profiles of *E. coli* in Poultry

**Figure 4:**
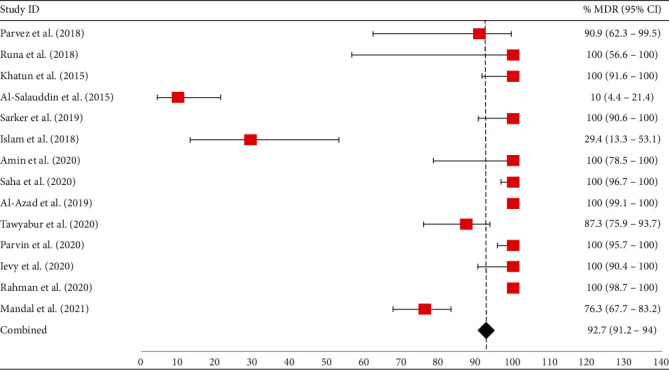
Forest plot of prevalence of MDR *E. coli* isolated from different poultry samples. The red-colored square dot represents the occurrence of MDR *E. coli* with their 95% confidence intervals, whereas the black-colored diamond-shaped point represents the combined MDR *E. coli* prevalence acquired from selected published articles between 2010 and 2021 in Bangladesh. The forest plot was created with Excel-365 after calculating prevalence and 95% confidence interval using GraphPad Prism software.

Out of 17 articles, 14 (82.4%, 95% CI: 58.9-93.8%) reported MDR *E. coli*. The occurrence of MDR *E. coli* ranged from 10% to 100%. Interestingly, 64.3% (9/14; 95% CI: 38.8-83.7%) of the articles recorded 100% (95% CI ranged from 56.6% to 100%) of MDR *E. coli* from poultry and poultry environment samples in Bangladesh. High levels of MDR *E. coli* were also detected from poultry and poultry environment samples, including 92.7% (95% CI: 91.2-94%), 90.9% (95% CI: 62.3-99.5%), 87.3% (95% CI: 75.9-93.7%), and 76.3% (95% CI: 67.7-83.2%) (Figure 4).

### 3.5. Genotypic Resistance Profiles of *E. coli* Sourced from Poultry in Bangladesh

About 58.8% (95% CI: 36.0-78.4%) of the articles reported antibiotic resistance genes, such as genes encoding resistance to beta-lactams (*bla*_TEM_, *bla*_CTX-M-1_, *bla*_CTX-M-2_, *bla*_CTX-M-9_, *bla*_OXA-1_, *bla*_OXA-47_, *bla*_SHV_, and *CITM*), tetracyclines (*tetA*, *tetB*, and *tetC*), sulfonamides (*sulI* and *sulII*), fluoroquinolones (*qnrB* and *qnrS*), colistin (*mcr1* and *mcr3*), aminoglycosides (*rmtB*), streptomycin (*aadA1*), gentamicin (*aac-3-IV*), erythromycin (*ereA*), trimethoprim (*dfrA1*), and chloramphenicol (*catA1* and *cmlA*) (Table 3). The prevalence of these resistance genes ranged from 1.2% to 100% (Table 3).

### 3.6. Prevalence of Extended-Spectrum Beta-Lactamase-Producing *E. coli* Sourced from Poultry in Bangladesh

The phenotypic extended-spectrum beta-lactamase- (ESBL-) producing *E. coli* isolates sourced from poultry and poultry environments were recorded by Parvez et al. [28] and Parvin et al. [40], detecting ESBL in 88% (95% CI: 70.0-95.8%) and 86.1% (77.2-91.8%) of the isolates. The genotypic ESBL-producing *E. coli* isolates were reported by Rahman et al. [42], detecting ESBL in 13.9% (95% CI: 10.8-17.8%) of the isolates. Several other studies found ESBL genes in *E. coli* from poultry and poultry environments (Table 3).

## 4. Discussion

### 4.1. Colibacillosis in Poultry

The colibacillosis syndrome, which is caused by avian pathogenic *E. coli* (APEC), is one of the most widespread infectious bacterial infections affecting chickens and other poultry. Chickens are continually exposed to *E. coli* through feces, water, dust, and the environment because *E. coli* are always present in the gastrointestinal tract of birds and spread widely in feces [44]. Colibacillosis in birds of all ages is a global problem that has a substantial financial impact on the poultry industry. Losses are mostly monetary because of excessive mortality and lower productivity of affected birds, especially during the late lay period and the peak egg production period [45]. Avian colibacillosis can cause a wide range of symptoms, some of which are listed as follows: lymphocytic depletion of the bursa and thymus, lymphocytic infiltration of the air sacs, pericarditis, perihepatitis, and acute, potentially fatal septicemia [41].

### 4.2. Transmission of *E. coli* in Poultry

**Figure 5:**
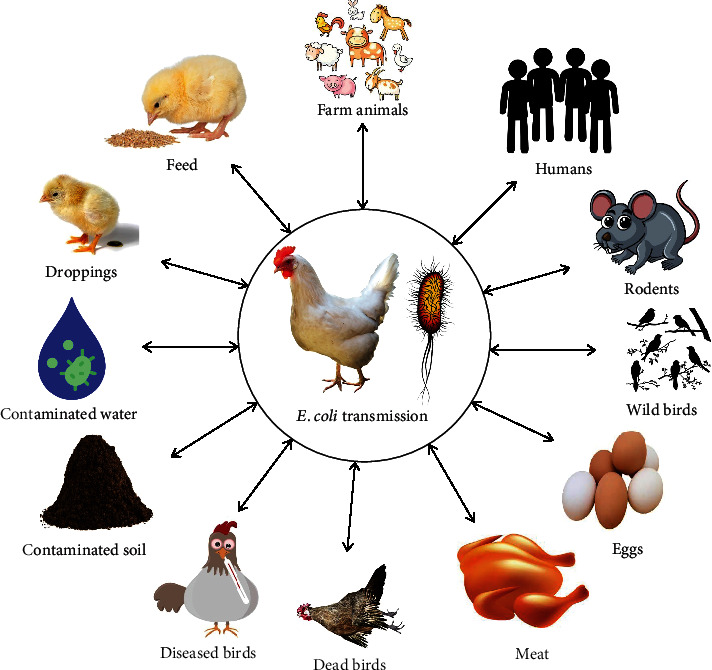
Possible transmission pathways of *E. coli* from different sources and vice versa.

The bacterium *E. coli* is a common inhabitant of the intestinal tract of chickens and other poultry species. Oral-fecal transmission between the same and different poultry species is possible [46]. It can reenter the environment in the feces of infected birds. Strains of *E. coli* that might cause various diseases are most likely to be found in the intestines and surroundings of chickens [47]. Poultry are most prone to contract *E. coli* infections (such as colibacillosis) through inhalation of infected dust. *E. coli* can be transmitted to new environments by a variety of vectors, including the darkling beetle, flies, insects, mites, rats, and wild birds [48]. *E. coli* transmission can occur horizontally and/or vertically, either directly or indirectly or both. Vertical transmission of *E. coli* can occur when a breeder carries the organism in their reproductive tract and then passes it on to their offspring [49].  Figure 5 depicts potential transmission pathways of *E. coli* infection poultry.

### 4.3. Prevalence of *E. coli* in Poultry

Our systematic review focused only on those studies which detected *E. coli* from both healthy and infected poultry and their environments using a PCR assay. The prevalence of *E. coli* ranged from 24.3% to 100%. Most of the articles (12/15) recorded a prevalence of more than 50% (61.7% to 100%). The combined prevalence of these studies was 69.3% (95% CI: 67.3-71.2%). The present review showed that poultry had a relatively high prevalence of *E. coli*. *E. coli* is a typical component of the gut microbiota in poultry because of being a characteristic occupant of the gastrointestinal tract. It can also be found in cloacal swabs, in the caecum, and in feces [50]. However, specific strains of *E. coli* can be the cause of colibacillosis, a frequent disease that affects poultry and is characterized by the infection of multiple organs in the bird, including the liver, kidneys, and spleen [41]. As a result, *E. coli* has been obtained from both the healthy and infected poultry populations. Moreover, the lack of hygienic maintenance on farms and their surroundings and the scarcity of proper knowledge among poultry farmers about an ideal poultry farming system play an important role in the higher prevalence of *E. coli* in poultry and poultry environments. The enhanced *E. coli* exposure in poultry and poultry environments indicates a threat to both poultry raising and human health. Humans can catch these diseases from undercooked meat and eggs, as well as from coming into contact with sick birds at the farm or slaughterhouse. Emerging problems in poultry health management and biosecurity pose a significant threat to zoonotic disease transfer to humans.

### 4.4. Antimicrobial Resistance Profiles of *E. coli* in Poultry

A worldwide epidemic of antibiotic resistance threatens human health in every way [51]. Many avian bacterial pathogens have developed antibiotic resistance as a result of the widespread use of antimicrobial agents for medicinal purposes and as growth-promoting agents to maintain increased growth and production in the poultry sector [52]. This systematic review revealed that *E. coli* sourced from poultry and poultry environments were resistant to 14 antimicrobial categories with 45 different antimicrobial agents. Interestingly, a recent study [40] reported that *E. coli* isolated from poultry meat showed resistance to 13 different classes of antibiotics, which is alarming to the healthcare communities.

According to the current review, *E. coli* isolates were highly resistant to the penicillin group of antibiotics, showing up to 100% resistance. The exhibition of higher resistance patterns of *E. coli* to the penicillin group of antimicrobials in poultry might be due to the longtime use of these antimicrobials, which indicates a cautious use of aminopenicillins for the treatment of *E. coli* infections in poultry. Moreover, *E. coli* isolates were resistant to the combinations of amoxicillin and clavulanate acid and piperacillin and tazobactam, which limits the antibiotic treatment options for *E. coli* infections. Because amoxicillin-clavulanate acid and piperacillin-tazobactam are usually used as alternatives to fluoroquinolone and carbapenem classes of antibiotics, respectively, to treat infections caused by extended-spectrum beta-lactamase- (ESBL-) positive *E. coli* isolates [53, 54].

Cephalosporins are a class of *β*-lactam antimicrobials that might be widely utilized as emergency drugs to treat important bacterial diseases in people and animals. Over a prolonged period, there was an increased record of resistance in *E. coli* to cephalosporin in humans and poultry. In Bangladesh, cephalosporins are frequently used in poultry for the treatment of *E. coli* infections. Based on our review, *E. coli* isolates showed up to 100% resistance to third-generation cephalosporins, which narrates a critical situation in antibiotic choice for the treatment of *E. coli* infections.

Fluoroquinolones are deemed first-line antibiotic therapy for *E. coli* infections [55]. Fluoroquinolones are widely used for the treatment of bacterial infections in humans, poultry, and other animals. Moreover, enrofloxacin is a typical fluoroquinolone that veterinarians prescribe to prevent early chick mortality and disease spread. But unfortunately, in the last decade in Bangladesh, *E. coli* isolates sourced from poultry were found to be highly resistant (up to 100%) to fluoroquinolones such as ciprofloxacin, levofloxacin, and nalidixic acid. These findings should serve as a warning regarding the rigorous application of fluoroquinolones in poultry production.

Colistin, under polymyxins, is a last-resort antimicrobial used for the treatment of human infections, but it is still widely used in intensive poultry production. In this review, *E. coli* isolates from poultry production in Bangladesh exhibited resistance to colistin (7.9%-100%). This finding of colistin resistance is quite alarming for the sake of public health in Bangladesh. However, the Bangladesh government has recently outlawed the production, sale, and dissemination of colistin and its derivatives for use in chicken production in an effort to combat colistin resistance. But we need to monitor and check up on the illegal use of colistin in poultry production regularly.

The carbapenem group of antibiotics includes ertapenem, imipenem, and meropenem. When treating a severe illness brought on by an ESBL-positive *E. coli*, carbapenems are occasionally the sole effective medication [56]. A carbapenem antibiotic, imipenem, has a diverse spectrum of antimicrobial effects on both aerobic and anaerobic bacteria. The rise of carbapenem-resistant bacteria poses a serious hazard to human health in light of the growing clinical usage of carbapenems. Our review indicates that carbapenems showed low to high resistance (13.6%-72.7%) in *E. coli* sourced from poultry and poultry environments. According to this high proportion of resistance of *E. coli* against carbapenems, the fact that antibiotics from the carbapenem group are widely employed as “last-line medicines” to treat illnesses brought on by MDR Gram-negative bacteria shows that we need to be worried [57]. Moreover, carbapenems are often regarded as last-resort antimicrobials for the treatment of hospitalized patients with various bacterial infections [57]. Since these antimicrobials are not permitted for use in the poultry sector, it is unknown how this kind of resistance has spread to chickens. It is crucial to establish quality control and confirmation methods for the poultry processing and production business since higher incidences of carbapenem resistance in chickens are quite concerning.

Aminoglycosides and tetracyclines are the most commonly used antimicrobial agents for the treatment of domestic animals, including poultry species [23]. Aminoglycosides are antimicrobials that inhibit the production of bacterial proteins [58]. Bangladesh has long utilized gentamicin, a broad-spectrum aminoglycoside antibiotic, to treat both Gram-negative and Gram-positive bacteria in chickens. Tetracycline is one of the antibiotics that is often used in veterinary medicine. *E. coli* resistant to tetracycline and aminoglycosides in poultry in Bangladesh has shown varying degrees of tetracycline resistance. But the emergence of higher resistance of *E. coli* against these classes of antibiotics (up to 100%) in poultry and poultry environments necessitates the use of legal alternative options for these antimicrobial categories for the betterment of poultry production.

Sulfa drugs, including sulfonamides and sulfamethoxazole, are a class of antimicrobials that are often used in poultry. Veterinarians frequently administer sulfa drugs to chickens as a therapeutic, preventative, or growth-promoting agent to prevent bacterial growth in poultry production [59]. However, inappropriate use of these antibiotics resulted in the increasing level of resistance of *E. coli* in poultry, which supports the current findings from our review. Based on our current review, *E. coli* in poultry and poultry environments were found to have varying degrees of sulfamethoxazole resistance in Bangladesh. Up to 100% resistance was found to sulfamethoxazole-trimethoprim, and more than 40% resistance to sulfonamides were observed in *E. coli* from poultry in different divisions of Bangladesh.

Azithromycin, under macrolides, is commonly used for the treatment of invasive *E. coli* infections, especially in humans, showing resistance (11.8%-34.9%) in *E. coli* isolates in poultry and poultry environments, which indicates a serious health issue. These azithromycin-resistant *E. coli* isolates have the potential to be transmitted to humans from poultry and poultry environments via direct and indirect contact. Moreover, *E. coli* isolates showed very high resistance (up to 100%) to another macrolide, erythromycin. The higher percentages of erythromycin resistance in *E. coli* isolates found in poultry are not unpredictable because of the wide range of use of this antibiotic in poultry production in Bangladesh.

Moreover, *E. coli* isolated from poultry and poultry environments showed resistance to other important antimicrobial categories, such as cephamycins, nitrofurans, monobactams, and glycylcyclines. Cephamycins and monobactams, along with fluoroquinolones and aminoglycosides, are recommended to use in the treatment of infections developed by ESBL-producing *E. coli* [60]. Nitrofurans were previously used in poultry production, but their use in food-producing animals was banned due to the presence of genotoxic and carcinogenic effects of these classes of antibiotics [61]. The resistance of *E. coli* against these classes of antibiotics in poultry in Bangladesh showed a serious issue in animals, humans, and the environment.

AMR is a problem for global public health, and when MDR strains appear, bacteria pose a serious threat to healthcare communities [62]. In this review, 14 out of 17 articles showed MDR *E. coli*; among them, the majority of the articles (9/17) reported 100% MDR *E. coli* in poultry and/or poultry environments. Moreover, the combined MDR of those articles was more than 90%. The excessive amounts of antimicrobial agents used as preventive treatment and/or growth promoters in poultry production might be the cause of this high percentage of MDR in *E. coli* isolates. In Bangladesh, according to a survey, about 80% of chicken producers utilized antibiotics as a prophylactic measure [31]. It is concerning that poultry producers employ overuse and misuse of antimicrobial agents in poultry production. In Bangladesh, antibiotics are easily available on the market and can be bought from those markets without any prescriptions or consultations from veterinarians. MDR bacteria might eventually take the place of antibiotic-sensitive organisms in the surroundings where antimicrobial agents are overly used [31].

Antimicrobial resistance genes were reported in 58.8% (10/17) of the published articles between 2010 and 2021. A wide range of resistance genes, e.g., genes encoding for beta-lactams, tetracyclines, fluoroquinolones, polymyxins, sulfa drugs, aminoglycosides, and phenicols, were detected in *E. coli* isolates sourced from poultry and poultry environments. Among them, at least one beta-lactam-associated resistance gene was reported in six articles and a tetracycline-associated resistance gene in five articles. All the resistance genes showed a higher percentage (up to 100%), indicating an alarming issue in the health system. Moreover, the detection of natural plasmids and transposons associated genes within the *E. coli* isolates (*tetA*, *tetB*, *tetC*, various *bla* genes, etc.) sourced from poultry and poultry environments indicates the plausible existence of multifarious genetic mobile elements [63].

Both phenotypic and genotypic ESBL-producing *E. coli* isolates were reported in poultry and poultry environments in Bangladesh. This review revealed that the ESBL-producing *E. coli* isolates were highly prevalent, harboring different types of ESBL genes. In many parts of the world, there has been an uptick in the emergence of ESBL-producing *E. coli* in both humans and animals [64]. ESBL-producing *E. coli* has been linked to the loss of effectiveness of multiple classes of antimicrobials, including tetracyclines, aminoglycosides, fluoroquinolones, and trimethoprim-sulfamethoxazole, all of which contribute to worsening healthcare outcomes, longer hospital stays, higher treatment costs, and more trouble keeping up with maintenance [14, 65, 66]. Therefore, the presence of ESBL-producing *E. coli* in poultry and poultry environments poses a great concern since it has given rise to a global crisis in the availability of antibiotic treatment options. Because ESBL-producing *E. coli* has the potential to be transferred to humans from poultry via direct and indirect pathways, their presence in poultry and poultry environments emerges a serious public health issue. Moreover, the alarmingly high prevalence of ESBL-producing *E. coli* calls for thorough risk assessments and targeted risk management to stem the tide of infections caused by these organisms.

These results from our systematic review highlight the need for an improved monitoring system and policies for the responsible use of antimicrobial drugs in Bangladesh's poultry industry. From the perspective of human health, this is a very promising outcome, as poultry owners can be introduced to antimicrobial-resistant zoonotic infections through interactions with their birds or the environment, or through consuming contaminated eggs or meat. Moreover, the results that have been obtained up to this point would be helpful in providing background information on antibiotic-resistant *E. coli* in order to prevent the spread of pan drug-resistant pathogens from animals to people.

### 4.5. Prevention and Control of *E. coli* Infections in Poultry

Reduced exposure to APEC and the consequences of stress and exacerbating diseases on avian susceptibility to APEC infection are two of the most important aspects of colibacillosis prevention. In addition, various commercial and trial immunizations can be used to prevent colibacillosis with varying degrees of efficacy. It is challenging to treat colibacillosis with antimicrobial therapy because of widespread MDR APEC and legal and public concerns about antimicrobial use in poultry. Most isolates are immune to treatments with sulfa, streptomycin, and tetracycline antibiotics. However, APEC's broad resistance to disinfectants, especially to some heavy metal compounds, makes it more challenging to control colibacillosis. Efforts to sterilize and clean the eggs laid by breeder flocks should be increased. Eggs should not be laid on the floor, and the hatchery should be cleaned up regularly. In order to lessen the likelihood of major diseases, chicken flocks should implement biosecurity measures and vaccination programs. Reducing ammonia and dust levels in barns may help reduce the environmental insult that *E. coli* often needs to infiltrate a flock.

### 4.6. Mitigations of AMR Issues in Poultry following One Health Approaches

**Figure 6:**
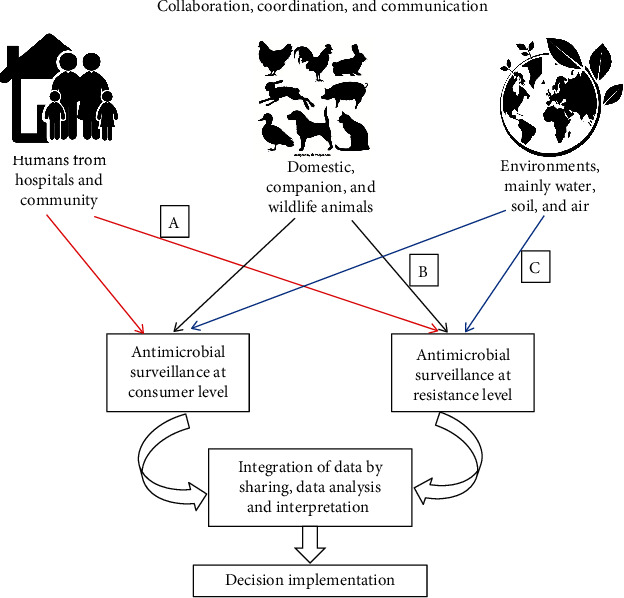
A framework of One Health surveillance program to mitigate AMR issues in poultry and other animals: (a) both commensal and clinical samples, (b) both commensal and clinical samples, and (c) commensal samples.

Since AMR affects human, animal, and environmental health, efforts to eradicate it must involve a concerted effort from a wide variety of sectors and stakeholders. The term “One Health” describes a strategy that encourages collaboration between healthcare providers. The One Health approaches may include the following:

- The importance of AMR as a danger to global health can be highlighted by rigorous surveillance efforts
- The primary goal is to improve both human and animal health by increasing the efficiency with which antimicrobial agents are used
- The use of antimicrobial agents at any level must be authorized by an expert veterinarian, human physician, and environmental specialist. The use of unnecessary antibiotics should be reduced at the animal, human, and environmental levels
- Knowledge of AMR and its repercussions among poultry farmers should be enhanced by implementing regular workshops on how to use antimicrobial agents properly and how to prevent and control different infectious diseases
- For effective risk management and policy action, knowledge of the prevalence of AMR in major foodborne pathogens and the prevalence of antibiotic residues in food and food products derived from animals is crucial
- Surveillance systems focusing on antimicrobial-resistant and pathogenically important microbial hazards in poultry should be implemented at the national and international levels. This surveillance includes (1) acquiring data at (data on antibiotic use) and after the consumer levels (data on AMR); (2) integration, analysis, and interpretation of acquired data; and (3) finally taking decision and implementation of action according to the outcomes of the surveillance (Figure 6).

### 4.7. Current Status and Future Research

Studies in Bangladesh during the previous decade found evidence of low-level epidemiological, antimicrobial resistance, and genetic research in detecting *E. coli* in poultry and poultry environments. The prevalence of food-borne illnesses in Bangladesh is higher than the global average, and further study is needed to determine why this happens. The following topics ought to be the center of future studies in order to obtain a better knowledge of the emergence of the AMR challenge and the ways in which to battle this public health danger in the context of Bangladesh and the wider world.

## 5. Conclusions

Our current systematic review revealed that *E. coli* sourced from poultry and poultry environments showed a higher resistance to almost all the antibiotic classes, indicating a serious health issue in all the communities. Although antimicrobial resistance is a concern for human health, this phenomenon has its origins in the interface between humans, animals, and wildlife, as well as the environment; as a result, resistant genes or bacteria find their way into the human food chain. It is important to pay attention to the fact that poultry production facilities in Bangladesh often fail to adhere to biosecurity, safety, and hygiene regulations. Cleanliness, proper manufacturing practices, and strict biosecurity are all crucial for preventing the spread of zoonoses and containing colibacillosis in poultry production facilities. There is an immediate necessity to fortify the awareness and scientifically based investigations via monitoring and surveillance program on AMR in order to minimize the hazardous effects of antimicrobial-resistant *E. coli* in poultry industries. Moreover, it needs a strong monitoring program at a national level to check the illegal use of antibiotics in poultry production.

## Figures and Tables

**Table 1:** Major findings of studies (2010–2021) focused on antimicrobial resistance in *E. coli* in poultry in Bangladesh.

Study duration/study area	Published year	Sample category	Sample types (*n*)	No. of PCR-positive *E. coli*	Resistance phenotype (DDT)	Resistance genotype (PCR)	MDR	ESBL	References
2012/Dhaka	2016	Broiler	Feces (40)	11	AMX, TE, STM, NFT, CIP, and LEV	*bla*_TEM_	√	DDST, PCR	[28]
2013/Jessore	2018	Broiler	Cloacal swabs (8)	5	AMP, CL, E, NEO, and P	—	√	—	[29]
2014/Sylhet	2015	Broiler	Cloacal swabs and liver sample (100)	42	GEN, E, P, CPX, AMX, and NA	—	√	—	[30]
2015/Mymensingh, Gazipur, and Sherpur	2015	Broiler	Dressed broiler (60)	50	AMX, AZM, CIP, E, GEN, NOR, S, and TE	—	√	—	[31]
2016/Chattogram	2019	Broiler	Cloacal swabs (60)	37	AMP, CRO, TE, STM, GEN, CL, C, CIP, NA, and E	*bla*_TEM_, *tet*A, and *sulII*	√	—	[32]
2017/Mymensingh	2018	Broiler	Cloacal swabs (65)	54	PLX, OFX, MOX, GAT, and LEV	*qnrS*	—	—	[33]
2017/Jamalpur, Tangail, Kishoreganj, and Netrokona	2018	Broiler	Dressed broiler (70)	17	AMX, AZM, E, GEN, NOR, S, and TE	—	√	—	[34]
2017/Chittagong	2020	Broiler	Cloacal swabs, environmental samples (300)	146	OXT, CIP	*tetA*, *tetB*, and *tetC*	—	—	[35]
2017-18/unknown	2020	Layer	Feces, cecum, poultry pen, and environment	104 isolates	AMP, TE, FOX, CRO, CTX, CAZ, CFM, FEP, CIP, NA, GEN, STM, NFT, and TAZ	*mcr-1*, *bla*_CTX-M-1_, *bla*_CTX-M-9_, *bla*_TEM_, *bla*_OXA-1_, *bla*_OXA-47_, *qnrB*, *qnrS*, and *rmtB*	√	PCR	[7]
2017-18/Narsingdi, Narayanganj, and Manikganj	2020	Layer	Droppings, cloacal swabs, internal organs, feed, water, and egg surface swabs	392 isolates	DOX, AMP, TE, NFT, CIP, NA, FOX, IMP, GEN, C, SUL, AZM, and Pb	—	√	—	[36]
2018/Dhaka and Rajshahi	2019	Broiler	Cloacal swabs (400)	400	AMP, TE, S, CIP, E, STM, CL, GEN, and LEV	*tet*A, *tet*B, *bla*_TEM_, *aadA1*, *ere*A, and *dfr*A1	√	—	[37]
2018/Mymensingh	2019	Broiler	Cloacal swabs (60)	44	ERT, MEM, IMP, and CL	*mcr-3*	—	—	[38]
2018-19/Mymensingh and Tangail	2020	Turkey	Feces and intestinal contents (55)	55	LEV, E, GEN, C, CIP, S, MEM, and TE	*tetA*	√	—	[39]
2019/Dhaka, Sylhet, Mymensingh, Chattogram, and Rajshahi	2020	Broiler	Frozen chicken meat (133)	86	CIP, NA, LEV, NOR, GAT, PLX, OFX, CPX, CE, CXM, CEC, CAZ, CRO, CTX, FEP, FOX, AMP, AMX, AMC, TAZ, IMP, MEM, CL, Pb, AZN, GEN, TOB, AMK, S, NEO, TE, OXT, DOX, STM, TIG, C, and AZM	*bla*_TEM_, *bla*_CTX-M-1_	√	DDST, PCR	[40]
2019/Mymensingh	2020	Layer	Feces, intestinal contents, and egg yolk, air (99)	82	AMP, TE, C, E, EN, NOR, CIP, S, CL, and GEN	—	√	—	[41]
2020/Sylhet, Moulvibazar, Sunamganj, and Habiganj	2020	Broiler and layer	Chicken meat swabs (600), broilers-300, and layers-300	381 (broilers-197, layers-184)	STM, E, TE, S, AMP, C, and GEN	*tetA sulI*, *aadA*1, *ereA*, *aac-3-IV*, *cmlA*, *catA1*, *bla*_SHV_, and *CITM*	√	PCR	[42]
2020/Mymensingh	2021	Broiler	Cloacal swab, farm sewage, and hand washes (150)	114	LEV, CIP, CAZ, CRO, CTX, AMC, CL, DOX, IMP, and MEM	—	√	—	[43]

*n* = total number of samples tested; DDT = disk diffusion test; PCR = polymerase chain reaction; MDR = multidrug resistance; AMX = amoxicillin; TE = tetracycline; STM = sulfamethoxazole-trimethoprim; NFT = nitrofurantoin; CIP = ciprofloxacin; LEV = levofloxacin; AMP = ampicillin; CL = colistin; E = erythromycin; NEO = neomycin; P = penicillin; GEN = gentamicin; CPX = cephalexin; NA = nalidixic acid; S = streptomycin; PLX = pefloxacin; OFX = ofloxacin; MOX = moxifloxacin; GAT = gatifloxacin; OXT = oxytetracycline; DOX = doxycycline; ERT = ertapenem; MEM = meropenem; IMP = imipenem; C = chloramphenicol; EN = enrofloxacin; NOR = norfloxacin; AZM = azithromycin; CTX = cefotaxime; CE = cephradine; CXM = cefuroxime; CEC = cefaclor; CRO = ceftriaxone; FOX = cefoxitin; CAZ = ceftazidime; CFM = cefixime; TAZ = tazobactam; SUL = sulfonamide; Pb = polymyxin B; FEP = cefepime; AMC = amoxiclav; AZN = aztreonam; TOB = tobramycin; AMK = amikacin; TIG = tigecycline; DDST = double-disk synergy test; ESBL = extended-spectrum beta-lactamase.

**Table 2:** Phenotypic antimicrobial resistance profiles of *E. coli* sourced from poultry in Bangladesh (articles published from 2010 to 2021).

Study ID (no. of isolates tested)	Name of antibiotics	No. of resistant *E. coli* (%)	95% CI
*Penicillins and beta-lactamase inhibitors*			
Parvez et al. [28] (11)	AMX	11 (100)	74.1-100
Runa et al. [29] (5)	AMP	5 (100)	56.6-100
	P	5 (100)	56.6-100
Khatun et al. [30] (42)	P	42 (100)	91.6-100
	AMX	42 (100)	91.6-100
Al-Salauddin et al. [31] (50)	AMX	40 (83.3)	71.9-90.7
Sarker et al. [32] (37)	AMP	37 (100)	90.6-100
Islam et al. [34] (17)	AMX	15 (88.2)	76.7-97.9
Amin et al. [7] (14)	AMP	14 (100)	78.5-100
	TAZ	4 (28.6)	1.7-54.7
Saha et al. [36] (114)	AMP	84 (73.7)	64.9-80.9
Al Azad et al. [37] (400)	AMP	400 (100)	99.1-100
Parvin et al. [40] (86)	AMP	77 (89.5)	81.3-94.4
	AMX	79 (91.9)	84.1-96.0
	AMC	36 (41.9)	32.0-52.4
	TAZ	61 (70.9)	60.6-79.4
Ievy et al. [41] (36)	AMP	36 (100)	90.4-100
Rahman et al. [42] (381)	AMP	377 (98.9)	97.3-99.6
Mandal et al. [43] (114)	AMC	23 (20.2)	13.9-28.5
*Cephalosporins*			
Khatun et al. [30] (42)	CPX	42 (100)	91.6-100
Sarker et al. [32] (37)	CRO	5 (13.5)	5.9-27.9
Amin et al. [7] (14)	CRO	14 (100)	78.5-100
	CTX	14 (100)	78.5-100
	CAZ	8 (57.1)	32.6-78.6
	CFM	13 (92.9)	68.5-99.6
	FEP	12 (85.7)	60.1-97.5
Parvin et al. [40] (86)	CPX	40 (46.5)	36.4-56.9
	CE	43 (50)	39.7-60.3
	CXM	37 (43)	33.1-53.6
	CEC	13 (15.1)	9.1-24.2
	CAZ	25 (29.1)	20.5-39.4
	CRO	2 (2.3)	0.4-8.1
	CTX	46 (53.5)	43.0-63.7
	FEP	62 (72.1)	61.8-80.5
Mandal et al. [43] (114)	CAZ	2 (1.8)	0.3-6.2
	CRO	9 (7.9)	4.2-14.3
	CTX	89 (78.1)	69.6-84.7
*Carbapenems*			
Saha et al. [36] (114)	IMP	26(22.8)	16.1-31.3
Sobur et al. [38] (44)	ERT	29 (65.9)	51.1-78.1
	MEM	19 (43.2)	29.7-57.8
	IMP	6 (13.6)	6.4-26.7
Tawyabur et al. [39] (55)	MEM	40 (72.7)	59.8-82.7
Parvin et al. [40] (86)	IMP	41 (47.7)	37.5-58.1
	MEM	36 (41.9)	32.0-52.4
Mandal et al. [43] (114)	IMP	75 (65.8)	56.7-73.9
	MEM	58 (50.9)	41.8-59.9
*Fluoroquinolones*			
Parvez et al. [28] (11)	CIP	9 (81.8)	52.3-96.8
	LEV	8 (72.7)	43.4-90.3
Khatun et al. [30] (42)	NA	42 (100)	91.6-100
Al-Salauddin et al. [31] (50)	CIP	3 (6)	1.6-16.2
	NOR	3 (6)	1.6-16.2
Sarker et al. [32] (37)	CIP	13 (35.1)	21.8-51.2
	NA	34 (91.9)	78.7-97.2
Mahmud et al. [33] (18)	GAT	7 (38.9)	20.3-61.4
	LEV	4 (22.2)	9.0-45.2
	MOX	10 (55.6)	33.7-75.4
	PLX	11 (61.1)	38.6-79.7
	OFX	10 (55.6)	33.7-75.4
Islam et al. [34] (17)	NOR	1 (5.98)	0.3-26.9
Das et al. [35] (97)	CIP	76 (78.4)	69.2-85.4
Amin et al. [7] (14)	CIP	12 (85.7)	60.1-97.5
	NA	12 (85.7)	60.1-97.5
Saha et al. [36] (114)	CIP	51 (44.7)	35.9-53.9
	NA	87 (76.3)	67.7-83.2
Al Azad et al. [37] (400)	CIP	400 (100)	99.1-100
	LEV	332 (83)	79.0-86.4
Tawyabur et al. [39] (55)	LEV	15 (27.3)	17.3-40.2
	CIP	37 (67.3)	54.1-78.2
Parvin et al. [40] (86)	CIP	38 (44.2)	34.2-54.7
	NA	53 (61.6)	51.1-71.2
	LEV	29 (33.7)	24.6-44.2
	NOR	37 (43)	33.1-53.7
	GAT	43 (50)	39.7-60.3
	PLX	76 (88.4)	79.9-93.6
	OFX	49 (56.9)	46.4-66.9
Ievy et al. [41] (36)	EN	20 (55.6)	39.6-70.5
	NOR	18 (50)	34.5-65.5
	CIP	18 (50)	34.5-65.5
Mandal et al. [42] (114)	LEV	93 (81.6)	73.5-87.6
	CIP	80 (70.2)	61.2-77.8
*Aminoglycosides*			
Runa et al. [29] (5)	NEO	5 (100)	56.6-100
Khatun et al. [30] (42)	GEN	42 (100)	91.6-100
Al-Salauddin et al. [31] (50)	GEN	3 (6)	1.6-16.2
	S	19 (38)	25.9-51.9
Sarker et al. [32] (37)	GEN	6 (16.2)	7.7-31.1
Islam et al. [33] (17)	GEN	2 (11.8)	2.1-34.3
	S	3 (17.7)	6.2-41.0
Amin et al. [7] (14)	GEN	10 (71.4)	45.4-88.3
Saha et al. [36] (114)	GEN	30 (26.3)	19.1-35.1
Al Azad et al. [37] (400)	S	400 (100)	99.1-100
	GEN	204 (51)	46.1-55.9
Tawyabur et al. [39] (55)	S	9 (16.4)	8.9-28.3
	GEN	9 (16.4)	8.9-28.3
Parvin et al. [40] (86)	GEN	7 (8.1)	3.9-15.9
	TOB	7 (8.1)	3.9-15.9
	AMK	15 (17.4)	10.9-26.8
	S	50 (58.1)	47.6-67.9
	NEO	24 (27.9)	19.5-38.2
Ievy et al. [41] (36)	S	7 (19.4)	9.8-35.0
	GEN	3 (8.3)	2.9-21.8
Rahman et al. [42] (381)	S	270 (70.9)	66.1-75.2
	GEN	105 (27.6)	23.3-32.3
*Tetracyclines*			
Parvez et al. [28] (11)	TE	11 (100)	74.1-100
Al-Salauddin et al. [31] (50)	TE	11 (22)	12.8-35.2
Sarker et al. [32] (37)	TE	37 (100)	90.6-100
Islam et al. [34] (17)	TE	3 (17.7)	6.2-41.0
Das et al. [35] (97)	OXT	97 (100)	96.2-100
Amin et al. [7] (14)	TE	14 (100)	78.5-100
Saha et al. [36] (114)	DOX	90 (78.9)	70.6-85.4
	TE	86 (75.4)	66.8-82.4
Al Azad et al. [37] (400)	TE	400 (100)	99.1-100
Tawyabur et al. [39] (55)	TE	29 (52.7)	39.8-65.3
Parvin et al. [40] (86)	TE	73 (84.9)	75.8-90.9
	OXT	80 (93)	85.6-96.8
	DOX	59 (68.6)	58.2-77.4
Ievy et al. [41] (36)	TE	36 (100)	90.4-100
Rahman et al. [42] (381)	TE	325 (85.3)	81.4-88.5
Mandal et al. [43] (114)	DOX	89 (78.1)	69.6-84.7
*Macrolides*			
Runa et al. [29] (5)	E	5 (100)	56.6-100
Khatun et al. [30] (42)	E	42 (100)	91.6-100
Al-Salauddin et al. [31] (50)	E	42 (84)	71.5-91.7
	AZM	6 (12)	5.6-23.8
Sarker et al. [32] (37)	E	6 (16.2)	7.7-31.1
Islam et al. [34] (17)	AZM	2 (11.8)	2.1-34.3
	E	12 (70.6)	46.9-86.7
Saha et al. [36] (114)	AZM	36 (31.6)	23.8-40.6
Al Azad et al. [37] (400)	E	400 (100)	99.1-100
Tawyabur et al. [39] (55)	E	55 (100)	93.5-100
Parvin et al. [40] (86)	AZM	30 (34.9)	25.7-45.4
Ievy et al. [41] (36)	E	35 (97.2)	85.8-99.9
Rahman et al. [42] (381)	E	341 (89.5)	86.0-92.2
*Polymyxins*			
Runa et al. [29] (5)	CL	5 (100)	56.6-100
Sarker et al. [32] (37)	CL	8 (21.6)	11.4-37.2
Amin et al. [7] (104)	CL	98 (94.2)	87.9-97.3
Saha et al. [36] (114)	Pb	9 (7.9)	4.2-14.3
Al Azad et al. [37] (400)	CL	106 (26.5)	22.4-31.0
Sobur et al. [38] (44)	CL	13 (29.6)	18.2-44.2
Parvin et al. [40] (86)	CL	9 (10.5)	5.6-18.7
	Pb	7 (8.1)	3.9-15.9
Ievy et al. [41] (36)	CL	4 (11.1)	4.4-25.3
Mandal et al. [43] (114)	CL	17 (14.9)	9.5-22.6
*Phenicols*			
Sarker et al. [32] (37)	C	10 (27)	15.4-42.9
Saha et al. [36] (114)	C	59 (51.8)	42.7-60.7
Tawyabur et al. [39] (55)	C	11 (20)	11.6-32.4
Parvin et al. [40] (86)	C	27 (31.4)	22.6-41.8
Ievy et al. [41] (36)	C	35 (97.2)	85.8-99.9
Rahman et al. [42] (381)	C	190 (49.9)	44.9-54.9
*Sulfonamides/folate pathway inhibition*			
Parvez et al. [28] (11)	STM	10 (90.9)	62.3-99.5
Sarker et al. [32] (37)	STM	35 (94.6)	82.3-99.0
Amin et al. [7] (14)	STM	12 (85.7)	60.1-97.5
Saha et al. [36] (114)	SUL	51 (44.7)	35.9-53.9
Al Azad et al. [37] (400)	STM	400 (100)	99.1-100
Parvin et al. [40] (86)	STM	76 (88.4)	79.9-93.6
Rahman et al. [42] (381)	STM	207 (54.3)	49.3-59.3
*Cephamycins*			
Amin et al. [7] (14)	FOX	3 (21.4)	7.6-47.6
Saha et al. [36] (114)	FOX	47 (41.2)	32.6-50.4
Parvin et al. [40] (86)	FOX	42 (48.8)	38.6-59.2
*Nitrofuran*			
Parvez et al. [28] (11)	NFT	3 (27.3)	9.8-56.6
Amin et al. [7] (14)	NFT	3 (21.4)	7.6-47.6
Saha et al. [36] (114)	NFT	72 (63.2)	54.0-71.5
*Monobactams*			
Parvin et al. [40] (86)	AZN	1 (1.2)	0.1-6.3
*Glycylcyclines*			
Parvin et al. [40] (86)	TIG	2 (2.3)	0.4-8.1

CI = confidence interval; AMX = amoxicillin; TE = tetracycline; STM = sulfamethoxazole-trimethoprim; NFT = nitrofurantoin; CIP = ciprofloxacin; LEV = levofloxacin; AMP = ampicillin; CL = colistin; E = erythromycin; NEO = neomycin; P = penicillin; GEN = gentamicin; CPX = cephalexin; NA = nalidixic acid; S = streptomycin; PLX = pefloxacin; OFX = ofloxacin; MOX = moxifloxacin; GAT = gatifloxacin; OXT = oxytetracycline; DOX = doxycycline; ERT = ertapenem; MEM = meropenem; IMP = imipenem; C = chloramphenicol; EN = enrofloxacin; NOR = norfloxacin; AZM = azithromycin; CTX = cefotaxime; CE = cephradine; CXM = cefuroxime; CEC = cefaclor; CRO = ceftriaxone; FOX = cefoxitin; CAZ = ceftazidime; CFM = cefixime; TAZ = tazobactam; SUL = sulfonamide; Pb = polymyxin B; FEP = cefepime; AMC = amoxiclav; AZN = aztreonam; TOB = tobramycin; AMK = amikacin; TIG = tigecycline.

**Table 3:** Genotypic antimicrobial resistance profiles of *E. coli* sourced from poultry in Bangladesh (articles published from 2010 to 2021).

Study ID	Detected resistance genes	*n*/*N*^∗^	% of AMR (95% CI)
Parvez et al. [28]	*bla*_TEM_	11/11	100 (74.1-100)
Sarker et al. [32]	*bla*_TEM_	28/37	75.7 (59.9-86.6)
	*tetA*	15/37	40.5 (26.4-56.5)
	*sulII*	13/35	35.1 (21.8-51.2)
Mahmud et al. [33]	*qnrS*	13/18	72.2 (49.1-87.5)
Das et al. [35]	*tetA*	20/20	100 (83.9-100)
	*tetB*	3/20	15 (5.2-36.0)
	*tetC*	2/20	10 (1.8-30.1)
Amin et al. [7]	*bla*_TEM_	10/14	71.4 (45.4-88.3)
	*bla*_CTX-M-1_	12/14	85.7 (60.1-97.5)
	*bla*_CTX-M-9_	1/14	7.1 (0.4-31.5)
	*bla*_OXA-1_	3/14	21.4 (7.6-47.6)
	*bla*_OXA-47_	2/14	14.3 (2.5-39.9)
	*mcr1*	14/14	100 (78.5-100)
	*qnrB*	2/14	14.3 (2.5-39.9)
	*qnrS*	4/14	28.6 (11.7-54.7)
	*rmtB*	4/14	28.6 (11.7-54.7)
Al Azad et al. [37]	*bla*_TEM_	365/400	91.3 (88.1-93.6)
	*tetA*	381/400	95.3 (92.7-96.9)
	*tetB*	381/400	95.3 (92.7-96.9)
	*aadA1*	353/400	88.3 (84.7-91.1)
	*ereA*	339/400	84.8 (80.9-87.9)
	*dfrA1*	262/400	65.5 (60.7-69.9)
Sobur et al. [38]	*mcr3*	7/13	53.9 (29.1-76.8)
Tawyabur et al. [39]	*tetA*	27/29	93.1 (78.0-98.8)
Parvin et al. [40]	*bla*_TEM_	86/86	100 (95.7-100)
	*bla*_SHV_	1/86	1.2 (0.1-6.3)
	*bla*_CTX-M-2_	1/86	1.2 (0.1-6.3)
Rahman et al. [42]	*sulI*	175/381	45.9 (40.9-50.9)
	*cmlA*	84/381	22.1 (18.2-26.5)
	*catA1*	27/381	7.6 (5.4-10.7)
	*ereA*	119/381	31.2 (26.8-36.1)
	*aac-3-IV*	94/381	24.7 (20.6-29.2)
	*tetA*	292/381	76.6 (72.1-80.6)
	*aadA1*	132/381	34.7 (30.0-39.6)
	*bla*_SHV_	38/381	9.9 (7.4-13.4)
	*CITM*	15/381	3.9 (2.4-6.4)

Here, CTX-M-1, CTX-M-2, and CTX-M-9 correspond to the groups of CTX-M enzymes. *n* = number of isolates showing positive to relevant resistance genes; *N*^∗^ = number of isolates tested; % = percentage of isolates showing positive to relevant resistance genes; AMR = antimicrobial resistance; CI = confidence interval.

## Data Availability

The datasets used and/or analyzed during the current study are available from the corresponding author upon reasonable request.
